# Determination of Dissociation Constants for the Interaction of Myosin-5a with its Cargo Protein Using Microscale Thermophoresis (MST)

**DOI:** 10.21769/BioProtoc.5176

**Published:** 2025-02-05

**Authors:** Rui Zhou, Jiabin Pan, Xiang-Dong Li

**Affiliations:** 1 Group of Cell Motility and Muscle Contraction, State Key Laboratory of Integrated Management of Pest Insects and Rodents, Institute of Zoology, Chinese Academy of Sciences, Beijing, China; 2 University of Chinese Academy of Sciences, Beijing, China

**Keywords:** MST, Dissociation constant, Protein interactions, Protein expression, Protein purification

## Abstract

Myosin-5a (Myo5a) is an actin-dependent molecular motor that recognizes a diverse range of cargo proteins through its tail domain, playing a crucial role in the transport and localization of various organelles within the cell. We have identified a new interaction between Myo5a and its cargo protein melanophilin (Mlph), i.e., the interaction between the middle tail domain of Myo5a (Myo5a-MTD) and the actin-binding domain of Mlph (Mlph-ABD), by GST pulldown assay. We then intend to obtain the dissociation constant between Myo5a-MTD and Mlph-ABD using isothermal titration calorimetry (ITC) or microscale thermophoresis (MST), both of which are two commonly used methods for determining quantitative data on protein interactions. The advantages of MST over ITC include less protein usage, shorter operation time, and higher sensitivity. In this protocol, we present a method for using MST to determine the dissociation constants of Myo5a-MTD and Mlph-ABD, which were purified through overexpression in bacteria using affinity chromatography. The dissociation constant values obtained directly reflect the binding strength between these two proteins and provide a foundation for the isolation and purification of the complex in the future.

Key features

• A protocol for determining the dissociation constants between two purified proteins using microscale thermophoresis (MST).

• Detailed procedures for purification of recombinant proteins expressed in *E. coli*.

## Background

Myosin-5a (Myo5a) is an actin-dependent unconventional myosin that primarily functions as a transporter within cells. Among the various cargo proteins associated with Myo5a, melanophilin (Mlph) is currently the most extensively studied [1]. Mlph is a scaffold protein that interacts with small GTPase Rab27a at the N-terminal portion and Myo5a at the C-terminal portion, thus bridging Rab27a and Myo5a to form a trimeric complex.

Previous research has confirmed that Mlph contains two domains that bind directly to Myo5a: Mlph-GTBD, known as the globular tail domain (GTD)-binding domain, which interacts with the GTD of Myo5a; and Mlph-EFBD, known as the exon-F binding domain, which interacts with melanocyte-specific exon-F in the Myo5a tail [2]. The discovery of a third Myo5a-Mlph interaction enhances our understanding of the role that Myo5a plays in melanosome transport. In our initial study, we employed the traditional GST pulldown assay to investigate protein interactions, which qualitatively demonstrated the interaction between Myo5a-MTD and Mlph-ABD. However, considering that Mlph employs multiple structural domains to simultaneously interact with Myo5a [3–5], the GST pulldown assay is insufficient for comparing the binding affinities of the different structural domains of Mlph with Myo5a. In contrast, MST is a highly sensitive technique that can determine dissociation constants for ligand proteins by measuring fluorescence changes in the target protein [6].

MST is a technique used to determine dissociation constants for ligand proteins by quantifying the thermophoretic movement of fluorescent molecules in response to a temperature gradient. This method typically requires only nanomolar concentrations of target proteins, allowing for high sensitivity in detecting interactions. Additionally, the determination of MST is not influenced by the buffer composition in the system [7]. Many MST protocols utilize the overexpression of eGFP proteins in the cells as target proteins [8]. While this approach is simple and efficient, it may impact the conformation of the target protein. In our biological protocol, we performed affinity assays using MST by fluorescently labeling the amino groups of GST-tagged Mlph-ABD, which was expressed in *E. coli* and purified in vitro, as the target protein. The His-tagged Myo5a-MTD, also expressed in *E. coli* and purified in vitro, served as the ligand protein. This strategy allows us to arbitrarily select two proteins as the target and ligand proteins and to freely adjust their concentration, facilitating the success of the experiment. However, our method has limitations. For instance, the proteins used in the experiment must be easily purifiable to avoid loss of fluorescent labeling. Additionally, the concentration of fluorescent dyes may become excessive after labeling, as proteins typically contain multiple amino groups.

## Materials and reagents

**Reagents**

1. IPTG (isopropyl β-D-1-thiogalactopyranoside) (Inalco, catalog number: 1758-1400)

2. Glutathione (GE Healthcare, catalog number: 17-5132-02)

3. Kanamycin (Inalco, catalog number: 1758-9316)

4. Ampicillin (Inalco, catalog number: 1758-9314)

5. Lysozyme (Solarbio, catalog number: L8120)

6. DNase I (Beijing DingGuoChangSheng, catalog number: DH113-5)

7. Glutathione-Sepharose 4 Fast Flow (GE Healthcare, catalog number: 17513201)

8. Ni-nitrilotriacetic acid agarose (QIAGEN, catalog number: 30210)

9. ATTO-488 NHS ester (ATTO-TEC GmbH, catalog number: AD488-35)

10. Coomassie brilliant blue G250 (Amresco, catalog number: M140-50G)

11. Tryptone (QiSong biology, catalog number: BQS133120)

12. Yeast extract (Oxiod, catalog number: LP0021)

13. Glucose (QiSong biology, catalog number: BQS119483)

14. Tris-base (Sigma, catalog number: T1387)

15. EDTA (Amresco, catalog number: 0105)

16. DTT (Amresco, catalog number: 0281)

17. β-Mercaptoethanol (Macklin, catalog number: M6230)

18. Imidazole (Sigma-Aldrich, catalog number: 0664)

19. Ethanol (Beijing Yili Fine Chemicals Co., Ltd)

20. Tween-20 (DingGuoChangSheng, catalog number: DH358-3)

21. DMSO (Leagene, catalog number: CC0118)

22. NaCl (Beijing Yili Fine Chemicals Co., Ltd)

23. MgCl_2 _(Beijing Yili Fine Chemicals Co., Ltd)

24. KCl (Beijing Yili Fine Chemicals Co., Ltd)

25. NaHCO_3 _(Beijing Yili Fine Chemicals Co., Ltd)

26. H_3_PO_4 _(Beijing Yili Fine Chemicals Co., Ltd)

**Solutions**

1. SOC (used for *E. coli* transformation) (see Recipes)

2. LB (used for *E. coli* culture) (see Recipes)

3. 10× TBS (used for equilibration of Ni-nitrilotriacetic acid-agarose or Glutathione-Sepharose 4 Fast Flow) (see Recipes)

4. GST-tag protein lysis buffer (used for GST-tagged protein purification) (see Recipes)

5. GST-tag protein washing buffer (used for GST-tagged protein purification) (see Recipes)

6. GST-tag protein elution buffer (used for GST-tagged protein purification) (see Recipes)

7. GST-tag protein dialysis buffer (used for GST-tagged protein purification) (see Recipes)

8. His-tag protein lysis buffer (used for His-tagged protein purification) (see Recipes)

9. His-tag protein washing buffer (used for His-tagged protein purification) (see Recipes)

10. His-tag protein elution buffer (used for His-tagged protein purification) (see Recipes)

11. His-tag protein dialysis buffer (used for His-tagged protein purification) (see Recipes)

12. Bradford (used for protein detection) (see Recipes)

13. MST buffer (used for dilution of fluorescently labeled proteins in the MST reaction) (see Recipes)

14. ATTO-488 NHS-ester dye (see Recipes)

**Recipes**

**1. SOC**

**Table:** 

Reagent	Final concentration	Quantity or Volume
Tryptone	2%	20 g
Yeast extract	0.5%	5 g
NaCl	0.05%	0.5 g
KCl	2.5 mM	0.83 mL
MgCl_2_	10 mM	10 mL
Glucose	20 mM	20 mL

Adjust the final volume to 1 L with distilled water.

**2. LB**

**Table:** 

Reagent	Final concentration	Quantity or Volume
Yeast extract	5 g/L	5 g
Tryptone	10 g/L	10 g
NaCl	10 g/L	10 g

Adjust the final volume to 1 L with distilled water.

**3. 10× TBS**

**Table:** 

Reagent	Final concentration	Quantity or Volume
Tris-base	0.5 M	60.57 g
NaCl	1.5 M	87.66 g

Adjust the final volume to 1 L with distilled water. Adjust pH to 7.5 with concentrated HCl.

**4. GST-tag protein lysis buffer**

**Table:** 

Reagent	Final concentration	Volume
Tris-HCl, pH 7.5	50 mM	4.5 mL
EDTA	2 mM	90 μL
DTT	1 mM	360 μL
Lysozyme		10 mg

Adjust the final volume to 90 mL with distilled water.

**5. GST-tag protein washing buffer**

**Table:** 

Reagent	Final concentration	Volume
TBS	1×	50 mL

Adjust the final volume to 500 mL with distilled water.

**6. GST-tag protein elution buffer**

**Table:** 

Reagent	Final concentration	Quantity or Volume
Tris-HCl, pH 8.0	50 mM	1 mL
NaCl	200 mM	1 mL
DTT	1 mM	20 μL
Glutathione	10 mM	0.06 g

Adjust the final volume to 20 mL with distilled water.

**7. GST-tag protein dialysis buffer**

**Table:** 

Reagent	Final concentration	Quantity or Volume
NaHCO_3_	130 mM	10.92 g
NaCl	50 mM	2.92 g

Adjust the final volume to 1 L with distilled water. Adjust pH to 8.2–8.3 with concentrated HCl.

**8. His-tag protein lysis buffer**

**Table:** 

Reagent	Final concentration	Volume
Tris-HCl, pH 7.5	50 mM	4.5 mL
EDTA	2 mM	90 μL
β-Mercaptoethanol	5 mM	30 μL
Lysozyme		10 mg

Adjust the final volume to 90 mL with distilled water.

**9. His-tag protein washing buffer**

**Table:** 

Reagent	Final concentration	Volume
Tris-HCl, pH 7.5	50 mM	20 mL
Imidazole, pH 7.5	15 mM	6 mL
NaCl	150 mM	15 mL
β-Mercaptoethanol	5 mM	140 μL

Adjust the final volume to 500 mL with distilled water.

**10. His-tag protein elution buffer**

**Table:** 

Reagent	Final concentration	Volume
Tris-HCl, pH 7.5	50 mM	1 mL
Imidazole, pH 7.5	250 mM	4 mL
NaCl	150 mM	750 μL
β-Mercaptoethanol	5 mM	5.6 μL

Adjust the final volume to 20 mL with distilled water.

**11. His-tag protein dialysis buffer**

**Table:** 

Reagent	Final concentration	Quantity or Volume
Tris-HCl, pH 7.5	10 mM	10 mL
NaCl	200 mM	50 mL
DTT	1 mM	1 mL

Adjust the final volume to 1 L with distilled water.

**12. Bradford**

**Table:** 

Reagent	Final concentration	Quantity or Volume
Coomassie brilliant blue G250	1.41 mM	100 mg
H_3_PO_4_	10% v/v	100 mL
Ethanol	5% v/v	50 mL

Adjust the final volume to 1 L with distilled water.

**13. MST buffer**

**Table:** 

Reagent	Final concentration	Volume
Tris-HCl, pH 7.8	50 mM	2.5 mL
NaCl	100 mM	1.25 mL
MgCl_2_	10 mM	0.5 mL
Tween-20	0.05% v/v	125 μL

Adjust the final volume to 50 mL with distilled water.

**14. ATTO-488 NHS-ester dye**

Dissolve 5 mg of ATTO-488 NHS-ester dye powder in 510 μL of DMSO. Aliquot into 20 μL per tube, lyophilize, and store at -80 °C. Each tube contains 0.196 mg of ATTO-488 NHS-ester.

**Laboratory supplies**

1. General-purpose dialysis bags 8000–14000 (for protein purification ) (ShangHai Yuye, catalog number: MD1425)

2. Gravity flow column B (for column chromatography) (NanoTemper Technologies GmbH, catalog number: L001)

3. 200 μL PCR tubes (for MST experiments) (Axygen, catalog number: PCR-02-C)

4. Monolith capillaries (for MST experiments) (NanoTemper Technologies GmbH, catalog number: MO-K002)

## Equipment

1. Ultrasonic cell pulverizer (for cell lysis) (NingBo XinZhi Biotechnology Co., LTD, model: JY92-II)

2. Ultracentrifuge (for rapid sample processing and preservation) (Beckman Coulter, model: Optima XPN-80)

3. Benchtop centrifuge (for precipitating insoluble proteins) (Eppendorf, model: 5415R)

4. Nanodrop-1000 (for measuring nucleic acid and protein concentrations) (Thermo Fisher)

5. NanoTemper^®^ Monolith NT.115 (for MST measurements) (NanoTemper Technologies GmbH)

## Software and datasets

1. Nanodrop-1000 4.64.0.0 (for determination of protein concentration) (Thermo Fisher Scientific)

2. Monolith^®^ NT.115 (for analyzing MST data) (NanoTemper)

3. KaleidaGraph 4.0 (for data analysis and graphing) (Synergy)

4. Illustrator CS6 (for drawing images) (Adobe)

5. Mo.Control software (for controlling MST experiments) (NanoTemper)

## Procedure

**A. Protein expression**

**Expression of GST-Mlph-ABD**

1. Transformation:

a. Transfer 100 ng of plasmids (1 μL) GST-Mlph-ABD/pGEX4T2 into 100 μL of BL-21(DE3) *E. coli* competent cells.

b. Keep the mixture on ice for 5 min, heat-shock at 42 °C for 45 s, and then immediately place back on ice for 2 min.

c. Add 200 μL of SOC medium to each sample and incubate at 37 °C with shaking for 45 min.

d. Spread 100 μL of the above culture to an ampicillin-resistant agar plate (LB agar with ampicillin) and incubate the plate at 37 °C for 12–16 h.

2. Culture of *E. coli*:

a. When the colonies on the plate grow to the appropriate size, pick up a single colony to inoculate 4 mL of the ampicillin-resistant LB medium (at room temperature) and incubate at 37 °C with shaking at 200 rpm for 6 h.

b. Use the above culture (~4 mL) to inoculate 250 mL of the ampicillin-resistant LB medium (room temperature) and incubate at 37 °C with shaking at 200 rpm for 3–4 h until the OD_600_ reaches 0.8–1.

c. Add 50 μL of 1 M IPTG (final concentration 0.2 mM) to the above culture to induce protein expression and incubate with shaking at 200 rpm at 37 °C for 3 h or at 17 °C for 12 h (General note 1).

3. Collect *E. coli*:

a. Harvest the induced *E. coli* by centrifugation at 4,000 rpm (~3,000× *g*) for 10 min at room temperature.

b. Resuspend the *E. coli* pellets with 1× TBS and precipitate the *E. coli* by centrifugation again at 4,000 rpm (~3,000× *g*) for 10 min at room temperature. Discard the supernatant and use the pellets for purification directly or store them at -80 °C for later use.

**Expression of His-tagged Myo5a tail (His-Myo5a-MTD and His-Myo5a-MTDΔG)**

1. Transformation:

a. Transfer 100 ng of bacterial expression plasmids (1 μL) His-Myo5a-MTD/pET30a or His-Myo5a-MTDΔG/pET30a into 100 μL of BL-21(DE3) *E. coli* competent cells (Myo5a-MTD can interact with Mlph, whereas Myo5a-MTDΔG cannot).

b. Keep the mixture on ice for 5 min, heat-shock at 42 °C for 45 s, and then immediately place back on ice for 2 min.

c. Add 200 μL of SOC medium to each sample and incubate at 37 °C with shaking for 45 min.

d. Spread 100 μL of the mixture to a kanamycin-resistant agar plate (LB agar with kanamycin) and incubate the plate at 37 for 12–16 h.

2. Culture of *E. coli*:

a. When the colonies on the plate grow to the appropriate size, pick up a single colony to inoculate 4 mL of the kanamycin-resistant LB medium (at room temperature) and incubate at 37 °C with shaking at 200 rpm for 6 h.

b. Use the above culture (~4 mL) to inoculate 250 mL of the kanamycin-resistant LB medium (room temperature) and incubate at 37 °C with shaking at 200 rpm for 3–4 h until the OD_600_ reaches 0.8–1.

c. Add 50 μL of 1 M IPTG (final concentration 0.2 mM) to the above culture to induce protein expression and incubate with shaking at 200 rpm at 37 °C for 3 h or at 17 °C for 12 h (General note 1).

3. Collect *E. coli*:

a. Harvest the induced *E. coli* by centrifugation at 4,000 rpm (~3,000× *g*) for 10 min at room temperature.

b. Resuspend the *E. coli* pellets with 1× TBS and precipitate the *E. coli* again by centrifugation at 4,000 rpm (~3,000× *g*) for 10 min at room temperature. Save the pellets and store at -80 °C or use them immediately for purification.

**B. Protein purification**

**Figure 1.**
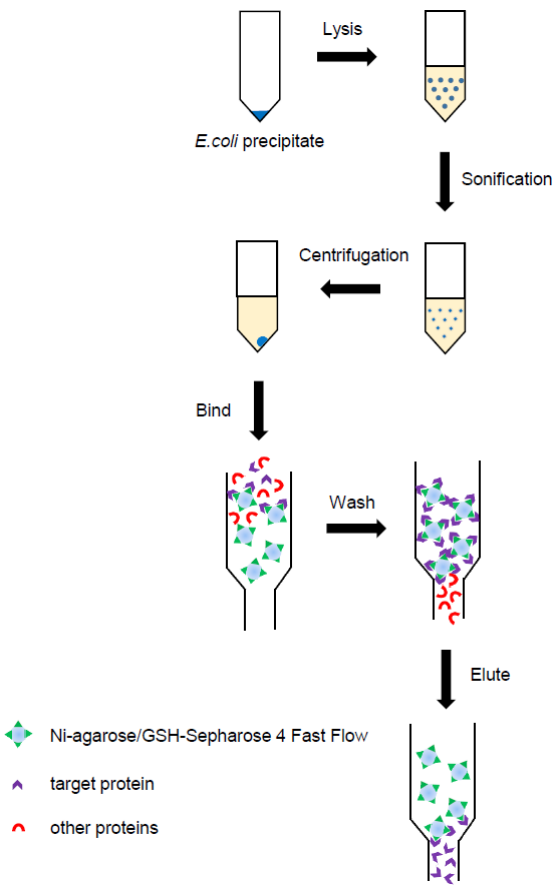
Flowchart for protein purification

The purification of proteins requires several sequential steps, as shown in Figure 1.

**For the purification of GST-Mlph-ABD**

1. Lysis:

a. Thaw the *E. coli*. pellet of GST-Mlph-ABD, which was collected from 250 mL of culture and stored at -80 °C.

b. Resuspend the *E. coli* pellets in 25 mL of GST-tag protein lysis buffer by pipetting up and down repeatedly and then stand on ice for 20 min.

c. Add 10^4^ U/mL DNase I to achieve a final concentration of 10 U/mL, 1 M MgCl_2_ to a final concentration of 3 mM, and 4 M NaCl to a final concentration of 0.2 M. Mix by inverting and leave on ice for 10 min.

2. Sonication: Set the power of the ultrasonic cell pulverizer to 200 W, with a cycle of 3 s on and 7 s off, repeating this process 80 times for complete lysis of *E. coli* (General note 2).

3. Centrifugation: Centrifuge the lysate at 20,000 rpm (~40,000× *g*) using an ultracentrifuge for 40 min at 4 °C and save the supernatant of the lysate in a 50 mL conical tube.

4. Binding to GSH-Sepharose 4 Fast Flow beads (GSH-Sepharose beads):

a. Equilibrate GSH-Sepharose 4 beads: Suspend 1 mL of GSH-Sepharose 4 beads in 10 mL of 1× TBS; then, let the beads settle down and discard the supernatant.

b. Transfer the GSH-Sepharose 4 beads to the 50 mL conical tube containing the supernatant of the lysate and rotate the conical tube at 4 °C for 2 h.

5. Wash:

a. Centrifuge the 50 mL conical tube at 2,000 rpm (~800× *g*) for 10 min at 4 °C using a multifunctional tabletop centrifuge.

b. Discard the supernatant, then suspend the GSH-Sepharose 4 beads with ~50 mL of GST-tag protein washing buffer and centrifuge again at 2,000 rpm (~800× *g*) for 10 min. Discard the supernatant.

c. Transfer the GSH-Sepharose 4 beads to a disposal chromatography column. Rinse the column with GST-tag protein wash buffer and detect protein in the eluate by mixing 5 μL of eluate with 45 μL of Bradford staining solution. The color of the Bradford staining solution changes from brown to blue when it reacts with protein. Stop rinsing the column when the eluate does not change the color of Bradford staining solution.

**Figure 2.**
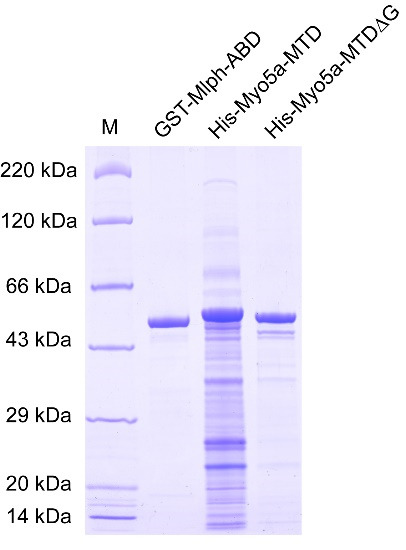
SDS-PAGE of purified GST-Mlph-ABD, His-Myo5a-MTD, and His-Myo5a-MTDΔG

6. Elution: Elute the target proteins using GST-tag elution buffer by gravity flow. Detect the proteins in the eluate by mixing 5 μL of eluate with 45 μL of Bradford staining solution. The color of the Bradford staining solution changes from brown to blue when it reacts with protein. Combine the eluate fractions containing high-concentration proteins.

7. Dialysis: Cut an appropriate length of the dialysis tube based on the estimated volume of the eluted protein. Place the dialysis tube in deionized water and boil at 100 °C for 3 min. Transfer the eluted proteins into the dialysis tube and dialyze against 1 L of GST-tag protein dialysis buffer overnight at 4 °C. This step is essential for labeling GST-Mlph-ABD with ATTO-488 NHS-ester, as it removes free amines.

8. Concentration:

a. Transfer the dialyzed proteins into ultrafiltration tubes and centrifuge at 4,000 rpm (~3,000× *g*) at 4 °C using a multifunctional tabletop centrifuge until the appropriate concentration is reached.

b. Aliquot the concentrated protein into a small volume (20–200 μL), quickly freeze in liquid nitrogen, and store at -80 °C.

c. Measure the protein concentration using Nanodrop-1000 and detect protein purity by SDS-PAGE (Figure 2). The purified GST-Mlph-ABD can be used for subsequent fluorescent labeling.

**Purification of His-tagged Myo5a tail (His-Myo5a-MTD and His-Myo5a-MTDΔG)**

1. Lysis:

a. Thaw E. coli pellets of His-Myo5a-MTD or His-Myo5a-MTDΔG, which were collected from 250 mL of culture and stored at -80 °C.

b. Resuspend the *E. coli* pellets in 25 mL of His-tag protein lysis buffer by pipetting up and down repeatedly and then stand on ice for 20 min.

c. Add 10^4^ U/mL DNase I to achieve a final concentration of 10 U/mL, 1 M MgCl_2_ to a final concentration of 3 mM, and 4 M NaCl to a final concentration of 0.2 M. Mix by inverting and leave on ice for 10 min.

2. Sonication: Set the power of the ultrasonic cell pulverizer to 200 W, with a cycle of 3 s on and 7 s off, repeating this process 80 times to fully lyse the organisms (General note 2).

3. Centrifugation: Centrifuge the lysate at 20,000 rpm (~40,000× *g*) using an ultracentrifuge for 40 min at 4 °C and save the supernatant of the lysate in a 50 mL conical tube.

4. Binding to Ni-nitrilotriacetic acid-agarose (Ni-agarose):

a. Suspend 1 mL of Ni-agarose in 10 mL of 1× TBS, let the beads settle down, and discard the supernatant.

b. Transfer the GSH-Sepharose 4 beads to the 50 mL conical tube containing the supernatant of the lysate and spin the conical tube on a rotary shaker at 4 °C for 2 h.

5. Wash:

a. Centrifuge the conical tube at 2,000 rpm (~800× *g*) for 10 min at 4 °C using a multifunctional tabletop centrifuge.

b. Discard the supernatant, then suspend the Ni-agarose with ~50 mL of His-tag protein washing buffer and centrifuge again at 2,000 rpm (~800× *g*) for 10 min. Discard the supernatant.

c. Transfer the Ni-agarose to a disposal chromatography column. Rinse the column with His-tag protein washing buffer and detect protein in the eluate by mixing 5 μL of eluate with 45 μL of Bradford staining solution. The color of Bradford staining solution changes from brown to blue when it reacts with protein. Stop rinsing the column when the eluate does not change the color of the Bradford staining solution.

6. Elution: Elute the target proteins using His-tag elution buffer by gravity flow. Detect the proteins in the eluate by mixing 5 μL of eluate with 45 μL of Bradford staining solution. The color of Bradford staining solution changes from brown to blue when it reacts with protein. Combine the eluate fractions containing high-concentration proteins.

7. Dialysis: Estimate the volume of the eluted protein and cut an appropriate length of the dialysis tube. Place the dialysis tube in deionized water and boil at 100 °C for 3 min. Transfer the eluted proteins into the dialysis tube and dialyze overnight at 4 °C in 1 L of His-tag protein dialysis buffer.

8. Concentration:

a. Transfer the dialyzed proteins into ultrafiltration tubes and centrifuge at 4,000 rpm (~3,000× *g*) at 4 °C using a multifunctional tabletop centrifuge until the appropriate concentration is reached.

b. Aliquot the concentrated protein into small volumes (20–200 μL), quickly freeze in liquid nitrogen, and store at -80 °C.

c. Measure the protein concentration using a Nanodrop-1000 and detect the protein purity by SDS-PAGE (Figure 2). The purified His-Myo5a-MTD and His-Myo5a-MTDΔG are directly used for MST experiments.

**C. Labeling of GST-Mlph-ABD with ATTO-488 NHS-ester**

1. Preparation of fluorescent dye: Dissolve 0.196 mg of ATTO-488 NHS-ester with 20 μL of DMSO to make a 10 mM solution, and then dilute to 1 mM by mixing 2 μL of ATTO-488 NHS-ester with 18 μL of GST-tag protein dialysis buffer.

2. Labeling:

a. Prepare 90 μL of 40 μM GST-Mlph-ABD protein by diluting GST-Mlph-ABD protein stock with GST-tag protein dialysis buffer.

b. Add 10.8 μL of 1 mM ATTO-488 NHS-ester dye to 90 μL of 40 μM GST-Mlph-ABD protein, resulting in a 3:1 molar ratio of ATTO-488 NHS-ester dye to GST-Mlph-ABD. Mix well.

c. Incubate for 1 h at room temperature in the dark (General notes 3–6).

3. Purification of the labeled protein:

a. Equilibrate the gravity flow column B with 3 mL of MST buffer three times.

b. Add a maximum of 500 μL of labeling reaction to the center of column B. Let the sample enter the column completely (when using less than 500 μL, adjust the volume to 500 μL using MST buffer after the sample has entered the column) and discard the flowthrough.

c. Add 2 mL of MST buffer to the center of column B and collect in 200–250 μL fractions.

**Figure 3.**
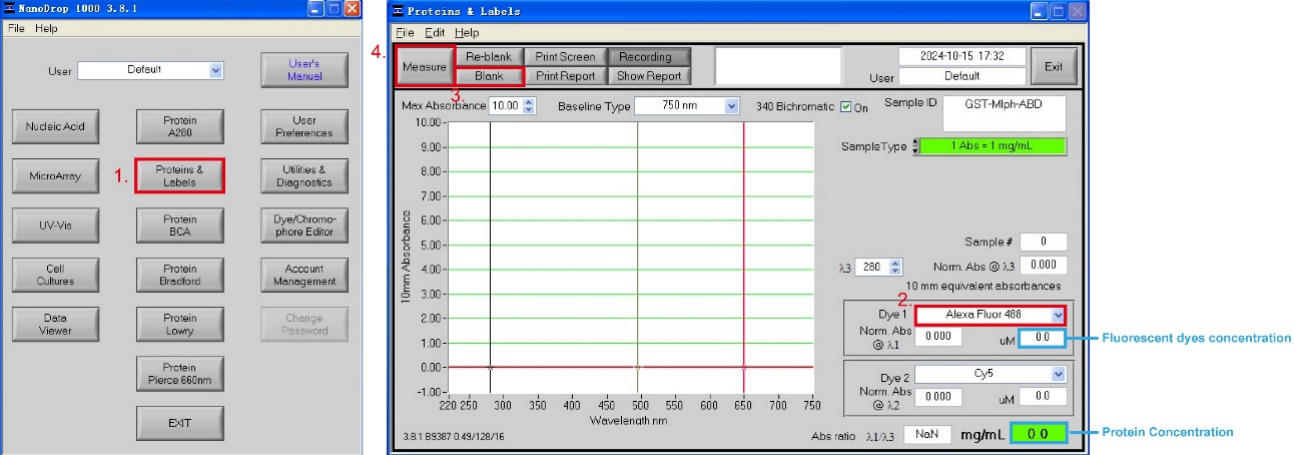
Measurement of the labeling efficiency of ATTO-488-labeled GST-Mlph-ABD using Nanodrop-1000

4. Measure ATTO-488-labeled GST-Mlph-ABD concentration in effluent fractions using Nanodrop-1000:

a. Add 3 μL of deionized water dropwise to the Nanodrop's detection probe. Open the software Nanodrop-1000 and select the module *Proteins & Labels*.

b. Select the excitation light *Alexa Fluor 488* for the fluorescent dye ATTO-488 NHS ester, add 3 μL of GST-tag protein dialysis buffer to the probe of the Nanodrop-1000, and click *Blank*.

c. Add 3 μL of effluent fractions to the Nanodrop-1000 probe and click *Measure*. The two blue highlight boxes in Figure 3 show the concentration of the fluorescent dye and protein.

d. Save the fractions containing high concentrations of ATTO-488-labeled GST-Mlph-ABD with a dye/protein molar ratio below 1.5 for subsequent MST experiments.

**D. MST measurement of the interaction between His-Myo5a-MTD and GST-Mlph-ABD**

**Figure 4.**
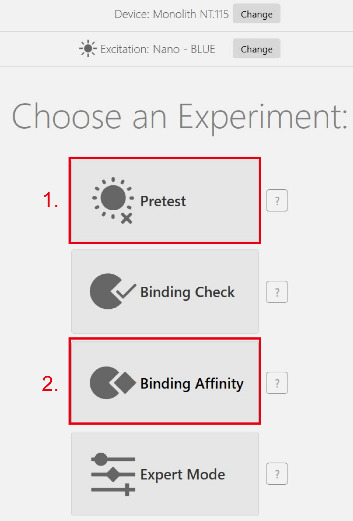
Initial setup of an experiment

1. Pre-test:

a. Dilute ATTO-488-labeled GST-Mlph-ABD to 40 nm with MST buffer.

b. Mix 10 μL of 40 nm ATTO-488-labeled GST-Mlph-ABD with 10 μL of His-tag protein dialysis buffer in a PCR tube, pipetting up and down several times to ensure adequate mixing, and then aspirate the mixture using Monolith capillaries (General note 7).

c. Put the capillaries in the slots on the sample tray, **not touching the optical measurement section of the capillary**.

d. Place the sample tray in the instrument by pushing it into the instrument tray slot as far as possible. The Monolith NT.115 instrument scans the tray and automatically determines the position of the capillaries on the tray.

e. Start the Mo.Control software and select *Start New Session*.

f. Select the green excitation filter for the upcoming experiment (General note 8).

g. Select the mode *Pretest* before performing a binding experiment and perform a pre-test experiment to detect the fluorescence intensity of the labeled GST-Mlph-ABD (see red highlight 1 in Figure 4). The fluorescence intensity of the target protein needs to be between 200 and 1,000, with a value between 400 and 800 being optimal.

2. Sample loading:

a. Take 16 clean PCR tubes, labeled 1–16, and arrange them in order on the PCR tube holder.

b. Add 10 μL of His-Myo5a-MTD dialysis buffer to tubes 2–16 and 20 μL of 40 μM His-Myo5a-MTD to tube 1.

c. Dispense 10 μL of His-Myo5a-MTD from tube 1 and transfer it into tube 2, mixing thoroughly. Then, using the same tip, transfer 10 μL from tube 2 into tube 3. Repeat this process until tube 16. After thoroughly mixing the sample in the final tube 16, remove 10 μL from that tube and discard it (General note 9).

d. Add 10 μL of ATTO-488-labeled GST-Mlph-ABD to each of the 16 tubes, mix well, and centrifuge the samples at 13,000× *g* for 5 min. Transfer the sample from the PCR tubes into 16 clean Monolith capillaries sequentially.

e. Put the capillaries in the slots on the sample tray (General note 10).

f. Place the sample tray in the instrument by pushing it into the instrument tray slot.

**Figure 5.**
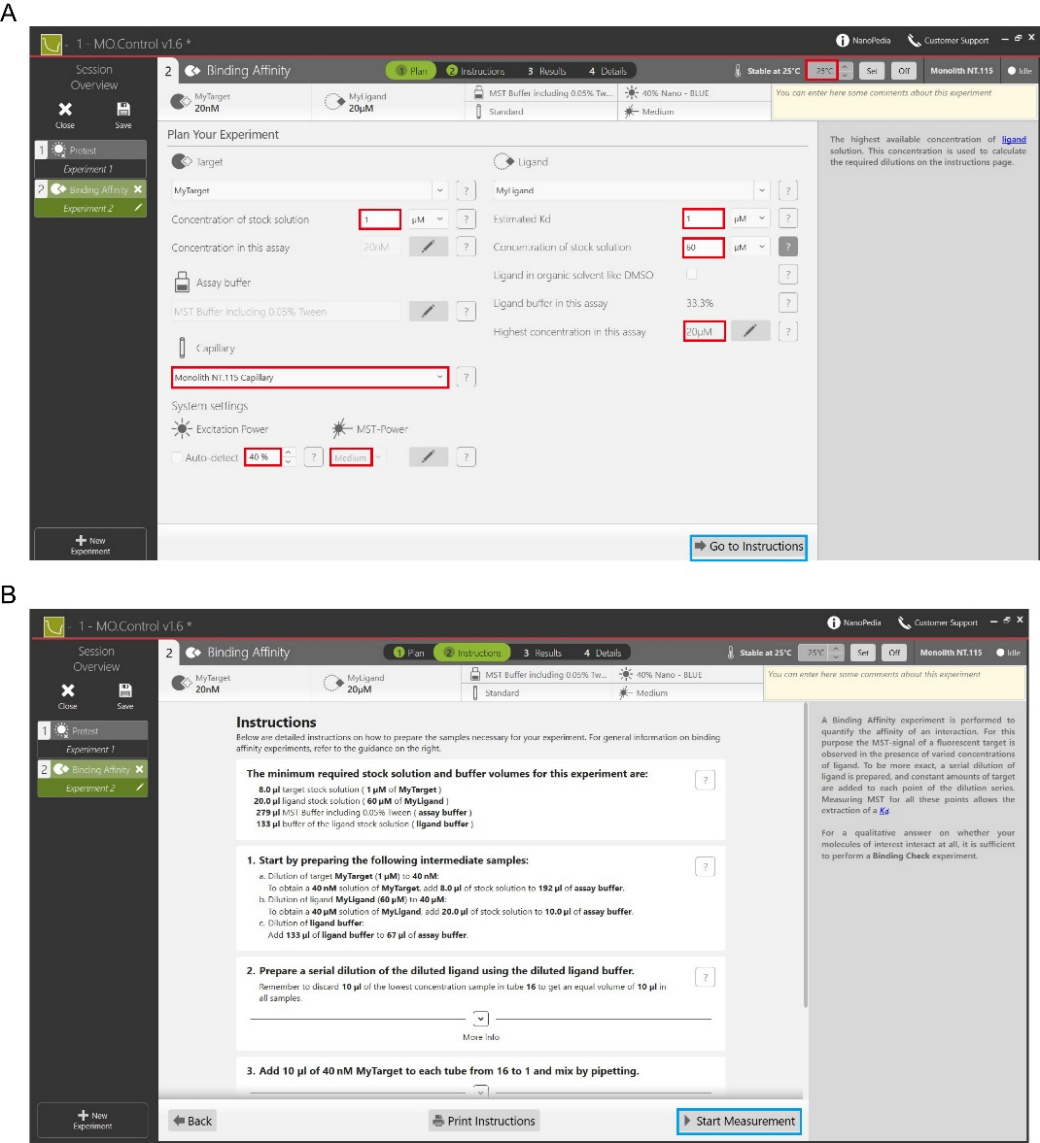
Procedure for measuring binding affinity experiments using Mo.Control software. (A) *Plan* interface for setting the temperature of the reaction, initial and final concentrations of the target and ligand proteins, excitation power, and MST power. (B) *Instruction* interface that provides a protocol on how to dilute the target and ligand proteins to the desired concentration for binding.

3. Binding affinity measurement:

a. Return to the homepage of the Mo. Control software and select *Binding Affinity* (see red highlight 2 in Figure 4).

b. Enter the *Plan* interface and set the following parameters: the reaction temperature of the MST experiment, the initial and final concentrations of the target and ligand proteins, the estimated Kd, the type of capillary, the excitation power and the MST power (see red highlight in Figure 5A).

c. Click *Go to Instructions* (see blue highlight in Figure 5A) to enter the *Instruction* interface, which allows you to dilute the target and ligand proteins to the appropriate concentrations according to the protocol provided.

d. Click *Start Measurement* to enter the *Results* interface (see blue highlight in Figure 5B) and wait for the system to automatically measure the MST traces and the dissociation constant Kd.

## Data analysis

The MST data can be analyzed with Monolith^®^ NT.115, which comes with the MST machine, or using third-party software. Here, we show how to analyze MST data using the KaleidaGraph 4.0 software.

**Figure 6.**
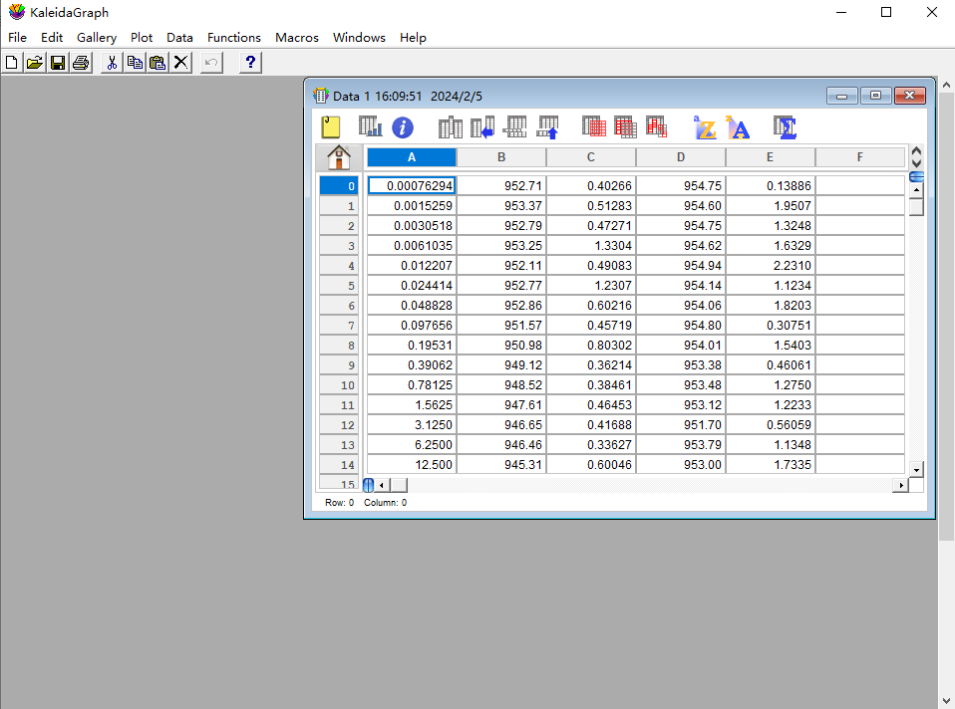
Screenshot of the KaleidaGraph version 4.0 software showing the location of the *Data* window under the *File* tab

1. Input data:

a. Open the KaleidaGraph software and select *File* in the first icon from the top row of the KaleidaGraph software (Figure 6).

b. Select *New* under the *File* menu to enter the MST experiment data separately into the Data 1 table. Dose values should be entered in column A. The mean values of response obtained from three repetitions of MST experiments for two different sets of interacting proteins are entered in columns B and D. Columns C and E correspond to the standard deviation of the mean values in B and D over three experiments, respectively.

**Figure 7.**
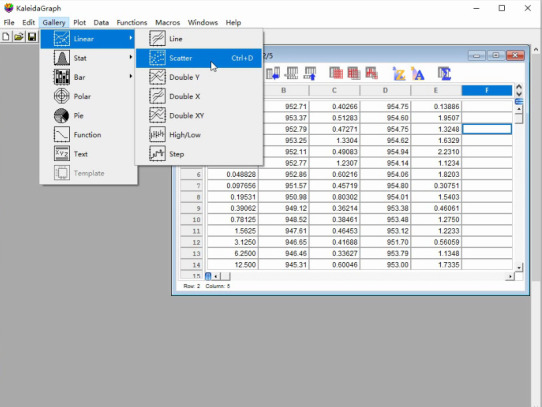
Screenshot of the KaleidaGraph version 4.0 software showing the submenu *Scatter* under the *Linear* menu under the *Gallery* tab

**Figure 8.**
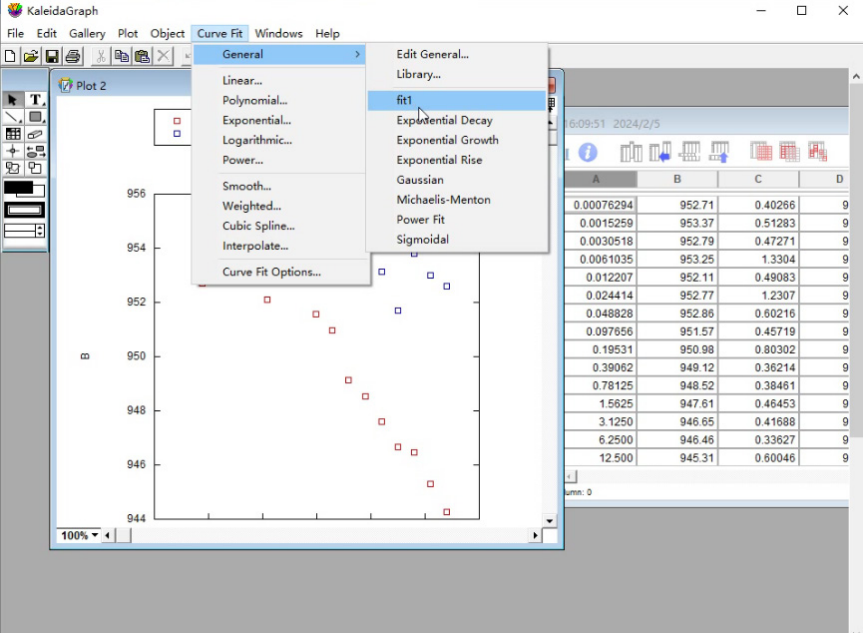
Screenshot of the KaleidaGraph version 4.0 software showing the submenu *fit1* under the *General* menu under the *Curve Fit* tab

**Figure 9.**
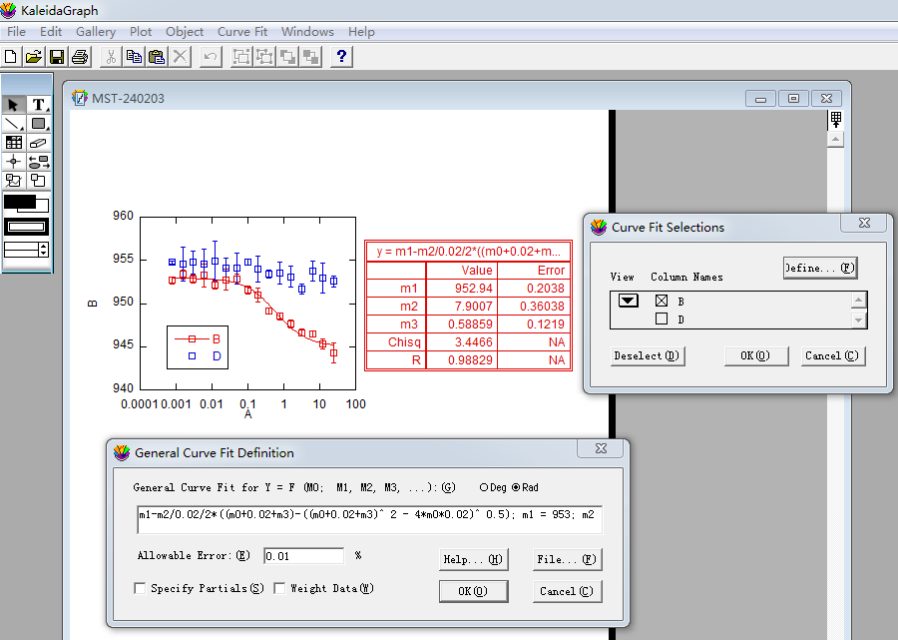
Screenshot of KaleidaGraph version 4.0 software showing the *Curve Fitting Selection* sub-window under the *Curve Fitting Selection* window

2. Analysis data:

a. Select *Gallery* in the third icon from the top row of the KaleidaGraph software (Figure 7).

b. Select *Scatter* under the *Linear* menu to generate a dot plot.

c. Select *Curve Fit* in the sixth icon from the top row of the KaleidaGraph software and choose *fit1* under the *General* menu (Figure 8).

d. Select the data in column B in the pop-up *Curve Fitting Selection* window. Click *Define* in the upper-right corner and enter the MST curve fitting formula (Figure 9):

m1 - m2/0.02/2*((m0 + 0.02 + m3) - ((m0 + 0.02 + m3) ^ 2 - 4*m0*0.02) ^ 0.5)/2; m1 = 953; m2 = 8; m3 = 0.5.

**Figure 10.**
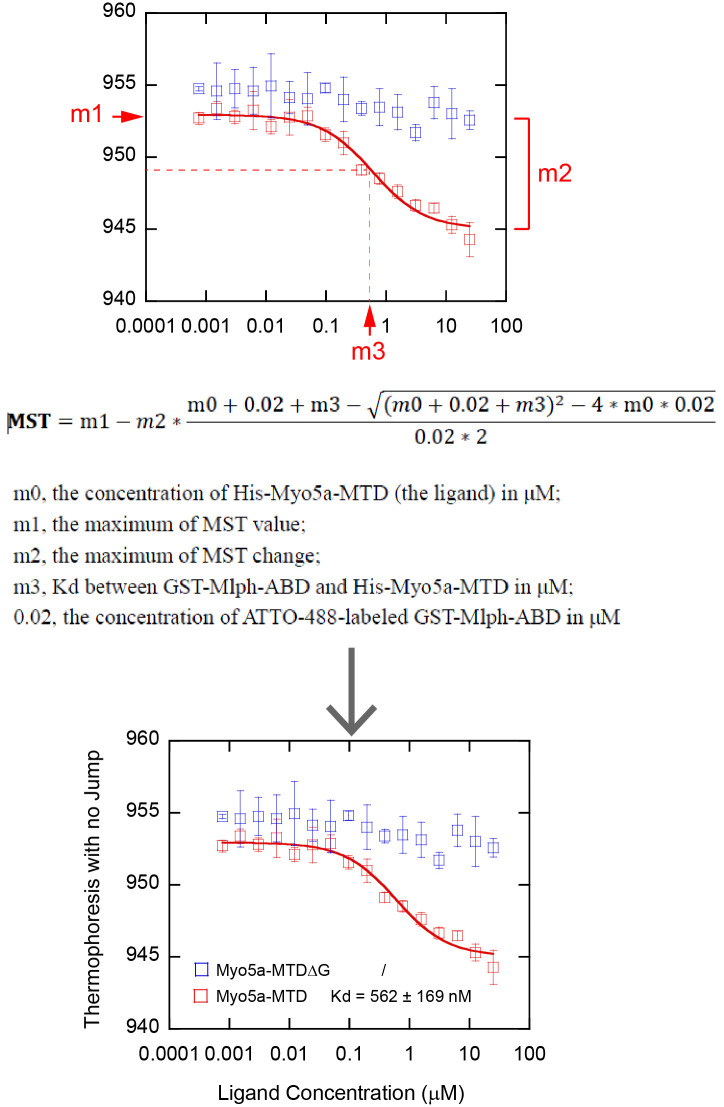
Analysis of MST data using KaleidaGraph. Top, MST data plots and curve fits using KaleidaGraph. Middle, curve fitting equation and interpretation of related parameters. Bottom, the final figure used in publication (Pan et al. [9], https://elifesciences.org/articles/93662).

Assign the value of m1 as the maximum MST value, m2 as the maximum MST change, and m3 as the estimated K_d_ value in μM, which is the x-value corresponding to the vertical coordinate reaching half of the maximum of MST change (Figure 10) (General note 11).

e. Click *File* and then *save Graph as* to save it in TIF format for editing in Illustrator CS6.

## Validation of protocol

This protocol or parts of it has been used and validated in the following research article:

• Pan et al. [9]. Identification of a third myosin-5a-melanophilin interaction that mediates the association of myosin-5a with melanosomes. *eLife* (Figure 3A).

We monitored the interaction between His-Myo5a-MTD and GST-Mlph-ABD using MST and obtained the dissociation constant (Kd) of 562 ± 169 nM of Myo5a-MTD for binding to Mlph-ABD. Consistent with the GST pulldown assay, we found that deletion of the C-terminal half of exon-G (His-Myo5a-MTDΔG) greatly decreased MST signaling.

## General notes and troubleshooting

1. The optimal conditions for IPTG to induce the expression of different proteins vary, and factors such as the concentration of IPTG, *E. coli* culture temperature, *E. coli* culture time, and stain of *E. coli* need to be taken into account. In general, a lower IPTG concentration can reduce the burden of protein expression on host cells, while a higher IPTG concentration can induce protein expression more rapidly. Low-temperature induction can prolong the time of protein expression so that the protein can be folded sufficiently, but it may also cause protein degradation, while the opposite is true for high-temperature induction. In practice, the optimal conditions should be determined based on experimental requirements to achieve the best protein expression results. We routinely use 0.2 mM IPTG to induce protein expression.

2. After sonication, it can be observed that the bacterial solution becomes transparent; if it is still turbid, it may be that the lysis is not sufficient, and the volume of lysis buffer or the number of sonication needs to be increased.

3. Avoid using buffers containing primary amines (e.g., ammonium ions, tris, glycine, ethanolamine, glutathione) or imidazole for labeling fluorescent proteins, which compete with the labeled proteins to reduce labeling efficiency.

4. Concerning reducing agents, DTT and β-mercaptoethanol interfere with the labeling reaction and, therefore, need to be avoided. If a reducing agent is required during the labeling reaction, use TCEP. However, DTT and β-mercaptoethanol are better suited than TCEP for the subsequent MST experiment, since TCEP may in some cases reduce reproducibility.

5. Purified proteins containing carriers like BSA will not be labeled properly and should not be used.

6. Labeled protein concentration: If the protein concentration is too low, the labeling efficiency will be greatly affected, and the general concentration will not be lower than 0.5 mg/mL.

7. Place the capillary horizontally into the reaction tube to aspirate the sample. Do not touch the capillary in the middle section where the optical measurement will be performed.

8. The excitation color can be changed at the start of each new experiment.

9. Thorough mixing ensures consistent fluorescence intensity of fluorescent proteins in each PCR tube.

10. Note the order of the capillaries. The highest concentration is placed in the front of the tray. This position is denoted as “1” on the sample tray and in the control software.

11. To fit the equations correctly, it is necessary to make approximately correct guesses about m1, m2, and m3.
